# Suicide attempts by adolescents assisted in an emergency department: a cross-sectional study

**DOI:** 10.1590/0034-7167-2022-0137

**Published:** 2023-04-07

**Authors:** Vanessa Dias Fogaça, Danton Mateus de Souza, Lucía Silva, Danila Maria Batista Guedes, Flavia Domingues, Isadora Trinquinato, Lisabelle Mariano Rossato

**Affiliations:** I Universidade de São Paulo. São Paulo, São Paulo, Brazil; II Universidade Federal de São Paulo. São Paulo, São Paulo, Brazil

**Keywords:** Suicide, Attempted, Adolescent, Suicide, Emergencies, Nursing., Intento de Suicidio, Adolescente, Suicidio, Urgencias Médicas, Enfermería., Tentativa de Suicídio, Adolescência, Suicídio, Emergência, Enfermagem.

## Abstract

**Objectives:**

to identify and characterize the care provided to adolescents admitted to an emergency department due to a suicide attempt.

**Methods:**

an observational, cross-sectional, descriptive study with a retrospective approach, carried out with medical records of adolescents aged 10 to 19 admitted for suicide attempts, between January 2015 and July 2020, in an emergency department. Data were subjected to descriptive and inferential analysis.

**Results:**

eighty-eight service occurrences were identified, mainly to females, exposed to multiple risk factors. Exogenous intoxication was the main method used, occurring at home and on weekdays. There were systemic repercussions, requiring multiple interventions and hospitalizations. Only 26% of cases were notified.

**Conclusions:**

adolescents treated for suicide attempts were exposed to multiple risk factors, with intoxication as the main means used. There is concern about the underreporting of cases and the logic of clinical care and medicalization.

## RESUMO

**Objetivos:**

identificar e caracterizar os atendimentos aos adolescentes admitidos em um departamento de urgência e emergência por tentativa de suicídio.

**Métodos:**

estudo observacional, transversal, descritivo, com abordagem retrospectiva, realizado com prontuários de adolescentes de 10 a 19 anos, admitidos por tentativa de suicídio entre janeiro de 2015 e julho de 2020 em um departamento de urgência e emergência. Os dados foram submetidos à análise descritiva e inferencial.

**Resultados:**

foram identificados 88 atendimentos, principalmente ao sexo feminino, expostos a múltiplos fatores de risco. A intoxicação exógena foi o principal meio utilizado, ocorrida no domicílio e em dias úteis. Houve repercussões sistêmicas, com necessidade de múltiplas intervenções e hospitalizações. Apenas 26% dos atendimentos foram notificados.

**Conclusões:**

os adolescentes atendidos por tentativa de suicídio estavam expostos a múltiplos fatores de risco, com a intoxicação como o principal meio utilizado. Preocupa a subnotificação dos casos e a lógica do cuidado clínico e medicalização.

## RESUMEN

**Objetivos:**

identificar y caracterizar la atención brindada a los adolescentes ingresados en un servicio de urgencias y emergencias por intento de suicidio.

**Métodos:**

estudio observacional, transversal, descriptivo, con enfoque retrospectivo, realizado con historias clínicas de adolescentes de 10 a 19 años, ingresados por intento de suicidio entre enero de 2015 y julio de 2020 en un servicio de urgencias y emergencias. Los datos fueron sometidos a análisis descriptivo e inferencial.

**Resultados:**

fueron identificadas 88 atenciones, principalmente del sexo femenino, expuestas a múltiples factores de riesgo. La intoxicación exógena fue el principal método utilizado, ocurriendo en el domicilio y entre semana. Hubo repercusiones sistémicas, requiriendo múltiples intervenciones y hospitalizaciones. Sólo el 26% de los casos fueron notificados.

**Conclusiones:**

los adolescentes asistidos por intento de suicidio estuvieron expuestos a múltiples factores de riesgo, siendo la intoxicación el principal medio utilizado. Preocupa el subregistro de casos y la lógica de atención clínica y medicalización

## INTRODUCTION

The World Health Organization (WHO) in 2014 defined suicidal behavior as a series of actions that include suicidal ideation, planning and attempt, and suicide itself. Suicidal ideation occurs when there is a desire to end one’s own life, but without any resulting action, it can be intertwined with the future planning of an action. Suicide attempt (SA), the focus of this study, is defined as any non-fatal suicidal behavior, such as exogenous intoxication, self-mutilation, self-inflicted injuries, which is linked to the intention to end one’s life, while suicide is the act of deliberately killing oneself^(1)^.

Worldwide, it is estimated that 703,000 people die by suicide each year, with an average of 80 suicides per 100,000 individuals. The WHO portrays that in 2019, for every 100 deaths, approximately 1.3% occurred due to suicide^(2)^. Brazil is included among the ten countries that record the highest absolute numbers of deaths by suicide^(3)^. After the implementation of compulsory notification in 2010, until 2016, Brazil reported 176,266 self-inflicted injuries, 27.4% of which were SA^(4-6)^. The data are alarming in the adolescent population, with suicide being the fourth leading cause of death in young people between 15 and 19 years of age, of both sexes^(2)^, concentrating on lowand middle-income countries in a proportion of 78% of this total, especially in countries with the highest rates of social inequality^(6)^. Despite the formulation of public policies and restructuring of the mental health care network, from 2006 to 2015, there was a 24% increase in suicides among adolescents in six large Brazilian cities^(7)^.

Regarding SA, in a systematic literature review with meta-analysis, it is observed that of 686,672 adolescents, 6% attempted suicide throughout their lives^(8)^. International data show that approximately 12% of adolescents experience suicidal ideation, 4% make a suicide plan and 4% attempt suicide^(9)^. However, the statistical data may be underestimated and be larger, due to the SA being underreported^(10)^.

SA tends to increase in adolescence, a unique stage of development, due to new experiences, imposed social demands and the construction of their personal identity. During this period, risk behaviors can be consolidated, with greater exposure to accidents and self-inflicted violence^(11)^. In a descriptive, qualitative, Brazilian study^(12)^, carried out in a Child Psychosocial Care Center, with 10 adolescents with a history of SA, it was observed that among the triggering reasons were: moving to another city and/or school; the fear and insecurity of making new friends; the loss of close family members; conflicting relationships and/or experiencing physical and/or psychological violence in a social and/or family environment; and difficulties in self-acceptance, factors that were accentuated in the context of the COVID-19 pandemic^(13)^. Faced with the challenges, they reported that suicide was seen as the only solution to problems, with the end of pain and sadness. Along the way, suffering was not easily relieved and there were multiple SA.

The international literature shows that for every 1,000 adolescents, between 15 and 19 years old, there are on average 4 to 5 visits to the emergency room per year due to SA. This search has increased by 92% in recent years, being an alarming fact that deserves attention^(14)^. As much as this first care is often performed in emergency contexts and has a clinical focus, WHO recommends that user embracement begin even in this context, as considering mental health needs can allow adolescents to consider the hospital service as a source of support and not as a fragmented service, with an eye only on the physical^(1)^.

Countless investigations have been dedicated to the study of suicidal behavior and its determinants, impacts, epidemiology and cultural variability, but focused on the primary and secondary level of health care in the territory^(10,12,15-17)^. As the hospital service plays a crucial role in identifying individuals at risk and/or assisting those who experience SA^(18)^, emergency departments can be particularly important for these efforts.

Thus, knowing the phenomenon of SA among adolescents in an emergency sector becomes necessary, due to the possibility of guiding formulations of holistic care strategies and encouraging the construction of suicide prevention policies, emerging and relevant phenomenon in the area of child and adolescent mental health. Thus, the following concern emerged: what is the profile and how is the care given to adolescents assisted in an emergency department due to SA?

## OBJECTIVES

To identify and characterize the care provided to adolescents admitted to an emergency department due to a SA.

## METHODS

### Ethical aspects

This study was approved by the Research Ethics Committees of the *Universidade de São Paulo* Nursing School and by the co-participating institution, 2018 and 2019, respectively. Data were collected upon approval of a Commitment Term. The ethical precepts of Resolution 466/12 and 510/16^(19)^ of the Brazilian National Health Council were respected.

### Study design, period and place

This is an observational, cross-sectional, descriptive study with a retrospective approach. To guide the methodology of this study, Strengthening the reporting of observational studies in epidemiology (STROBE) was used for cross-sectional studies^(20)^. The study was carried out in a secondary public hospital in the city of São Paulo. Retrospective data were collected from 2015 to 2020, a time frame determined from the availability of documents at the co-participating institution, to which medical records prior to 2015 were archived externally, depending on a different logistics for access. Medical records of adolescents assisted in the sectors of Child Emergency Room (CER), which serves children from the first days of life to adolescents up to 14 years 11 months and 29 days, and Adult Emergency Room (AER), were used. with adolescents between 15 and 19 years 11 months and 29 days and adults.

### Study population, inclusion and exclusion criteria

The sample consisted of individuals aged 10 to 19 years, 11 months and 29 days, classified as adolescents by the WHO^(1)^. Records of consultations between January 2015 and July 2020 were used, made available for collection by the institution’s Medical Archive and Statistics Service (SAME - *Serviço de Arquivo Médico e Estatística da instituição*).

Initially, we included adolescents, with the reason for care being considered according to the International Statistical Classification of Diseases and Related Health Problems 10^th^ Revision (ICD-10)^(21)^ as SA (X60 to X84), from January 2015 to July 2020. However, given the scarcity of records coded exclusively in this category (only 28 visits were registered as SA, ICD X60-84), it was decided to expand the classifications that were related to the means used for the SA, such as exogenous intoxication and violent accidents with undetermined intent. Thus, ICD X60 to X84 (self-inflicted violence), T50.9 to T65.9 (exogenous intoxications of undetermined intent) and Y09 to Y34 (events of undetermined intent) were adopted as new inclusion criteria.

It is noteworthy that, among the data analysis, to be considered SA, adolescents’ intention to take their own life was taken into account. To this end, the main researcher carried out a thorough reading of the documents that made up the medical record in search of any record of the service that mentioned intentionality and/or SA. For instance: a service to a teenager with exogenous intoxication, which during the anamnesis some member of the team documented that he used it with the intention of ending his own life, thus the service was considered as SA. Records that did not verify the intention of suicide were excluded.

### Study protocol

In order to obtain the service data, SAME was asked to select all medical records, whose reason for service fit the inclusion criteria. In the established period, 133 documents were made available to the researcher, namely: 1) Emergency service form, for people who were assisted for up to 24 hours; 2) Service envelopes for those who stayed for more than 24 hours; and 3) Medical records for subjects who had hospitalization. After a thorough reading of documents, as well as verification of suicide intentions, 88 medical records were selected to integrate the study population, 28 of which were classified as SA, and 60 records of service categorized by ICD T50.9 to 65.9 and Y09 to Y34.

For data collection, an instrument was elaborated by the researchers contemplating data referring to admission to the emergency service; characterization of assisted adolescent, their family and social context, previous assistance related to SA, professional assistance and SA itself; and mandatory notification.

### Analysis of results, and statistics

Data were stored in Microsoft Excel^®^, sent for statistical analysis using Microsoft^®^ SPSS version 22.0. Descriptive and inferential analyzes were performed, continuous variables were organized descriptively, categorical variables by absolute and relative frequency. The variables were associated using Pearson’s Chi-squared, Fisher’s exact, Wilcoxon-Mann Whitney, Brunner-Munzel, Wilch’s test and Student’s test. A statistical significance level of 5% was adopted (p<0.05).

## RESULTS

Eighty-eight adolescents with SA were included: 83% were female, mean age was 16.4 years (SD 2.29); 98.8% were single; 87.5% lived with their parents; 79.5% were students; 40.9% had a previous mental health problem documented in their medical records, with depression prevailing (31.8%); and 29.5% used daily medication (Table 1).

**Table 1:** Characterization of participating adolescents, São Paulo, São Paulo, Brazil, 2021

Adolescents assisted due to suicide attempts (N = 88)		
	**n (%)**	**Mean (min-max)**
Age (years)		16.4 (10.42-19.92)
Sex		
Female	73 (83.0)	
Male	15 (17.0)	
Color		
White	72(81.8)	
Brown	13 (14.8)	
Black	2 (2.3)	
Yellow	1 (1.1)	
Housing context		
Family members	77 (87.5)	
Friends	5 (5.7)	
Spouse	1 (1.1)	
Not reported	5 (5.7)	
Marital status		
Single	87 (98.9)	
Married	1 (1.1)	
Occupation		
Student	70 (79.5)	
Unemployed	4 (4.5)	
Informal work	2 (2.4)	
Not reported	12 (13.6)	
Previous mental health problem:		
Depression	28 (31.8)	
Anxiety	6 (6.8)	
Others	2 (2.3)	
Previous medication use		
Yes	26 (29.5)	
No	62 (70.5)	

### Care characterization

Thus, 31.8% of adolescents were treated at the CER and 68.2% at the AER. Service occurrences were classified as follows: 30.7% in ICD X60-84 (self-inflicted violence) and 69.3% in ICD T50-65.9 (exogenous intoxications of undetermined intent). The highest number of visits occurred in 2018 (26%), prevailing on weekdays (86.0%), morning hours (43.2%) and at home (83.0%).

Adolescents were taken to the emergency department, predominantly by their relatives (64%), with drowsiness (28%), nausea and vomiting (15.0) and confusion (12.0), with a Glasgow scale with score an average of 14.25. They underwent invasive procedures, such as an oro/nasogastric tube (35.2%), gastric lavage and intravenous (IV) rehydration in 34.1%. A total of 18.1% were admitted to the institution, mainly in the Intensive Care Unit (ICU) (11.3%), both pediatric (6.8%) and adult (4.5%). Table 2 below brings data regarding the initial service.

**Table 2:** Characterization of suicide attempt care in the pediatric emergency department, São Paulo, São Paulo, Brazil, 2021

Suicide attempt care characterization		
	**n (%)**	**Mean (SD)**
Year of care		
2015	17 (19.0)	
2016	13 (15.0)	
2017	13 (15.0)	
2018	15 (17.0)	
2019	23 (26.0)	
2020	7 (8.0)	
Care days		
Working days	75 (86.0)	
Weekend	13 (15.0)	
Care period		
Morning (7 a.m.- 12 p.m.)	38 (43.2)	
Afternoon (1 p.m.- 6 p.m.)	16 (18.2)	
Night (7 p.m.- 6 a.m.)	38 (43.2)	
Suicide attempt place		
Home	73 (83.0)	
School	4 (4.5)	
Public thoroughfare	2 (2.3)	
Not reported	9 (10.2)	
Transportation to the sector		
Family members	56 (64.0)	
Emergency mobile care service	14 (16.0)	
Transfer	3 (3.0)	
Others	10 (11.0)	
Not reported	5 (6.0)	
Clinical conditions^*^		
Drowsiness	48 (28.0)	
Nausea and vomiting	25 (15.0)	
Confusion	21 (12.0)	
Crying	20 (12.0)	
Agitation	16 (9.0)	
Epigastralgia	15 (9.0)	
Altered vital signs	10 (6.0)	
Headache	8 (5.0)	
Bleeding	7 (4.0)	
Glasgow score		14.25 (1.86)
Interventions carried out^*^		
Probe	31 (35.2)	
Gastric lavage	30 (34.1)	
Intravenous rehydration	30 (34.1)	
Activated charcoal	21 (23.9)	
Antidote	13 (14.7)	
Adolescent destination		
Wards	6 (6.8)	
Pediatric Intensive Care Unit	6 (6.8)	
Adult Intensive Care Unit	4 (4.5)	
Home	43 (48.9)	
Transfer	20 (23.0)	
Evasion	9 (10.0)	

* *More than one intervention performed on the same adolescent.*

Recurrence was found in 10 (6.9%) of the consultations, with four adolescents who sought care repeatedly. The mean time (MT) elapsed between SA and admission to the CER and AER was 6.11 hours (SD=11.15). These consultations had 11.29 hours (SD=20.60) as MT of permanence in the emergency department. For adolescents who were admitted to the department and discharged from hospital, the MT of stay was 13.07 hours (SD=10.58); in those who were transferred to another service, MT was 14.92 hours (SD= 16.89). In the 16 admissions, the average stay was 142.19 hours of hospitalization. The overall MT of hospitalization among all consultations was 36.79 hours (SD=63.87). Compulsory AS notification was filled out only in 26.1% of cases, concentrated (78.3%) in the years 2019 and 2020.

### Suicide attempt characterization

The method most frequently used by adolescents was exogenous intoxication, mainly by medication (84.1%). Thus, 7.9% of adolescents combined the use of two methods for SA, namely: drug intoxication and illicit drug intoxication; poison poisoning and illicit drug intoxication; illicit drug and alcohol intoxication; drug intoxication and precipitation; alcoholic intoxication and stab wounds. It is worth noting that marijuana, crack, cocaine, heroin, LSD and ecstasy were considered illicit drugs.

Among drug intoxications as a method of SA, the use of the following pharmacological groups was observed: antidepressants (28.4%), analgesics and antipyretics (20.5%), benzodiazepines (19.3%), non-steroidal anti-inflammatory drugs (15.9%), hypertension (12.5%), antiepileptics (11.3%), antipsychotics (10.2%), antihistamines (6.8%) and others (22.7%). 38 (43.2%) situations were identified with a mixture of two (52.6%), three (29.0%) and four (18.4%) drugs. As for who owned the medications used intentionally in excess, antidepressants, in 16 (64%) records, belonged to the adolescents themselves, as well as in eight (89%) reports of antipsychotic intakes and in seven (41.2%) of benzodiazepines. As for drugs such as analgesics, anti-inflammatories, antibiotics, systemic arterial hypotension and the other various drugs reported, they were mostly used by family members (p<0.001). In the case of antidepressants (p=0.001) and antipsychotics (p=0.018), there is a correlation with the excessive use of these drugs by users who already used them before SA. It is noteworthy that in the case of poisoning, the use of a pesticide popularly known in Brazil as “*chumbinho*” (dark gray granular substance, which contains aldicarb and other insecticides) was responsible for 100% of the cases, reported in eight cases (9%).

It is observed that drug intoxications are more frequent in females (p<0.001) and alcoholic intoxications in males (p=0.03). Stab wounds were means used only by male adolescents (p=0.02) as well as precipitation (p=0.02). Drug intoxication was more prevalent in older adolescents, with a mean age of 19.33 years (SD=0.35) (p<0.001). Table 3 below brings data regarding the means used by adolescents for AS, associated with age and sex.

**Table 3:** Characterization of the means used for the suicide attempt associated with age and sex variables, São Paulo, São Paulo, Brazil, 2021

Means used^*^	Categorical variable	Age			Sex		
					Female	Male	*p* value
		n (%)	Mean (SD)	*p* value	n (%)	n (%)	
Drug intoxication	Yes	74 (84.1)	16.3 (2.1)	0.113	67 (91.8)	7 (46.7)	<0.001
No	14 (15.9)	17.0 (3.0)	6 (8.2)	8 (53.3)
Alcohol poisoning	Yes	5 (5.7)	17.7 (3.1)	0.107	2 (2.7)	3 (20.0)	0.033
	No	83 (94.3)	16.3 (2.2)		71 (97.3)	12 (80.0)	
Illicit drug poisoning	Yes	4 (4.6)	19.3 (0.4)	<0.001	2 (2.7)	2 (13.3)	0.133
	No	84 (95.4)	16.3 (2.3)		71 (97.3)	13 (87.7)	
Poison	Yes	8 (9.1)	80 (16.4)	0.717	5 (6.9)	3 (20.0)	0.108
	No	80 (90.9)	16.4 (2.2)		68 (93.2)	12 (80.0)	
White weapon injury	Yes	2 (2.3)	19.1 (1.1)	0.089	0 (0.0)	2 (13.3)	0.027
	No	86 97.7)	16.3 (2.3)		73 (100)	13 (86.7)	
Precipitation	Yes	2 (2.3)	18.5 (1.7)	0.157	0 (0.0)	2 (13.3)	0.027
	No	86 (97.7)	16.4 (2.3)		73 (100)	13 (86.7)	

* *More than one medium used by the same adolescent.*

No description of risk factors was found in eight consultations (4%), either current or past. In those that contained a description, the main risk factors were observed: mental health problem (40.9%), previous attempt (39%), family conflict (39%) and self-mutilation (25%). Being experiencing or having experienced two associated risk factors were identified in 32% of cases, three in 16% and four factors in 11%.

As for risk factors in relation to age, it was observed that younger adolescents had a statistically significant relationship with the following risk factors: history of self-mutilation (p=0.02), bullying (p<0.01), physical abuse (p=0.02), family conflicts (p<0.001), living with separated parents (p<0.001), living with someone close who attempted suicide (p<0.001). Belonging to the LGBTQIA+ group and frequent use of alcohol were associated with male adolescents (p<0.001). Table 4 below shows the risk factors found in adolescents with SA and their associations with age and gender.

**Table 4:** Risk factors of adolescents with suicide attempts and association with age and sex variables, São Paulo, São Paulo, Brazil, 2022

Risk factor^*^	Categorical variable	Age			Sex		
					Female	Male	*p* value
		n (%)	Mean (SD)	*p* value	n (%)	n (%)	
Previous suicide attempt	Yes	34 (39.0)	16.6 (2.0)	0.693	27 (37.0)	7 (46.7)	0.485
No	54 (61.0)	16.3 (2.5)	46 (63.0)	8 (53.3)
Self-mutilation	Yes	22 (25.0)	15.4 (2.1)	0.020	18 (24.7)	4 (26.7)	0.870
	No	66 (75.0)	16.7 (2.3)		55 (75.3)	18 (24.7)	
Bullying	Yes	7 (8.0)	14.2 (1.8)	0.011	7 (9.6)	0 (0.0)	0.597
	No	81 (92.0)	16.6 (2.2)		66 (90.4)	15 (100)	
Physical abuse	Yes	2 (2.2)	12.1 (1.4)	0.025	2 (2.7)	0 (0.0)	1.000
	No	86 (97.8)	16.5 (2.2)		71 (97.3)	15 (100)	
Psychological abuse	Yes	5 (6.0)	14.7 (2.8)	0.083	5 (6.9)	0 (0.0)	0.583
	No	83 (94.0)	16.5 (2.2)		68 (93.2)	15 (100)	
Sexual abuse	Yes	5 (6.0)	15.9 (3.2)	0.593	3 (4.1)	2 (13.3)	0.199
	No	83 (94.0)	16.4 (2.3)		70 (95.9)	13 (86.7)	
Lesbian, Gay, Bisexual and Transgender	Yes	6 (7.0)	17 (2.2)	0.593	2 (2.7)	4 (26.7)	<0.001
	No	82 (93.0)	16.4 (2.3)		71 (97.3)	11 (73.3)	
Family conflict	Yes	34 (39.0)	15.4 (2.2)	<0.001	28 (38.4)	6 (40.0)	0.905
	No	54 (61.0)	17.1 (2.2)		45 (61.6)	9 (60.0)	
Love conflict	Yes	18 (20.0)	17.2 (2.1)	0.084	13 (17.8)	5 (33.3)	0.177
	No	70 (80.0)	16.2 (2.3)		60 (82.2)	10 (66.7)	
Frequent use of alcohol	Yes	12 (14.0)	17.4 (2.0)	0.124	7 (9.6)	5 (33.3)	0.015
	No	78 (86.0)	16.3 (2.3)		66 (90.4)	10 (66.7)	
Separated parents	Yes	12 (14.0)	14.3 (1.9)	<0.001	10 (13.7)	2 (13.3)	0.970
	No	76 (86.0)	16.7 (2.2)		63 (86.3)	13 (86.7)	
Suicide attempt of someone close	Yes	3 (3.0)	14.6 (0.5)	<0.001	2 (2.7)	1 (6.7)	0.433
	No	85 (97.0)	16.5 (2.3)		71 (97.3)	14 (93.3)	
Grief	Yes	3 (3.0)	15.4 (3.3)	0.464	2 (2.7)	1 (6.7)	0.433
	No	85 (97.0)	16.4 (2.3)		71 (97.3)	14 (93.3)	
Low school performance	Yes	7 (8.0)	15 (3.0)	0.472	5 (6.9)	2 (13.3)	0.400
	No	81 (92.0)	16.5 (2.2)		68 (93.1)	13 (86.7)	

* *More than one medium used by the same adolescent.*

Regret for SA was recorded in the documentation of 21.6% of consultations. Psychiatric assessment records and maintenance of previous treatment were verified in 10.3%. In the other cases, 31.1% were referred for psychotherapy, 27.6% started using psychiatric medication, prescribed by the department’s medical team, concomitantly with psychotherapy, and psychiatric hospitalization in one patient. For the rest, no specific treatment for the mental health problem was documented.

## DISCUSSION

In this study, 88 SA occurred by adolescents over a period of five years and six months, with 40.9% having previous mental health problems. Numerous risk factors have been documented, mainly associated with younger age. SA often occurred at home, on weekdays, in the morning, with 64% being taken to the service by family members. The service resulted in 18.1% admissions to the institution. The most commonly used method was exogenous intoxication, mainly by family use drugs or previous use by adolescents, more common in females, while the most violent methods were used by male adolescents. Compulsory notification of self-inflicted violence was carried out in only 26.1% of cases.

The international literature demonstrates that the search for assistance in emergency services is a source of shame for adolescents and their families^(22-23)^. When they occur, in a small portion, they are those who experience systemic repercussions, as in this study with drowsiness, nausea, vomiting, confusion and the like. This low search for the service is a consequence of the triple taboo of SA, experienced by the general population: 1) Taboo of death, with an intrinsic distance from discussions that refer to the phenomenon and when they are faced with losses, they live a masking of grief with difficulty in express emotions; 2) Taboo of suicide, phenomenon with extreme social stigma, which arouses ambivalent reactions such as shock, sadness and questioning before the act and at the same time guilt, judgments and anger towards the individual; and 3) Taboo of child and adolescent suicide, which adds to the ambivalent emotional reactions to children’s and adolescents’ social representation, who socially represent the kind and angelic figure and, when exposed to the phenomenon, hurt the social imagination’s expectation^(23)^.

SA care in emergency rooms is objective, with a focus on maintaining life, with multiple interventions and in the clinic, as listed in this study. Although adolescents remain for a few hours in the service, their psychological demands are often not addressed^(14-18)^, for demanding dedication, active listening and bonding, challenges to the emergency professional due to interruptions and intercurrences inherent to the activity. Moreover, it also requires training, security in the approach and service structure, new barriers faced, mainly due to the fragmentation of care by age between the child and adult department, as a result of public mental health policies^(10)^.

In 2017, the WHO reiterated that worldwide there is a shortage of specialist and/or trained professionals to work in SA, in addition to the lack of investments in mental health^(24)^, a fact that permeates all levels of care. In the hospital institution, both its architecture and the professional body are constituted and conceived for life promotion, an admission of SA can provoke innumerable reactions. In a Brazilian qualitative investigation, carried out with health professionals in two emergency departments, the description of “false SA”, with a character of dubious intentionality, carried out with the objective of “calling attention”^(25)^. This study illustrates the triple taboo^(23)^ that permeates among professionals. It is worth mentioning that adolescents intrinsically distance themselves from the health service, either due to personal phenomena or due to the low implementation of public policies in this age group, and when SA occurs, due to the negative professional social representation, they may feel “a burden”, have their assistance harassed, increasingly distance themselves from assistance^(10,12)^, accumulate psychic suffering and be exposed to new SA.

Primary and secondary care allows health professionals to work closely and longitudinally, unlike the reality of emergency care. However, in a descriptive qualitative study, carried out with the multidisciplinary teams of a Psychosocial Care Center and a Family Health Strategy, it is observed that professionals have knowledge about SA, but most have never provided assistance to an adolescent exposed to it, although it is expected that these services are familiar with the subject. Moreover, in the speeches, judgment and professional taboo are noted^(10)^.

In this study, 86% of SA occurred on weekdays, in the morning (83%) and at home (83%). This finding may be related to the lower possibility of adult supervision, probably due to work activities, in addition to the fact that school activities take place on these days, exposing them to situations that can precipitate suffering and impulse, such as bullying, associated in this study with self-mutilation (p=0.02). It is noted that in 64% of cases adolescents were found and referred to the emergency department by their relatives, who are also experiencing impacts and must be welcomed.

The adolescent’s impulsive behavior can awaken in the family a feeling of impotence due to the loss of control over their actions, with the experience of a painful process of guilt and anger. Common statements are: “I never imagined that this could happen”, “How did I not realize that something was not right?”^(26)^. This family nucleus must be addressed within its uniqueness, with help in starting over with affective marks of a SA, lacking care and prevention strategies^(23)^.

In this study, the most used method for SA was exogenous drug intoxication (84.1%). In the case of adolescents, especially younger ones, the notion of toxicity, poisoning and overdose effects of some medications may be inaccessible, which can be a protective or dangerous factor, with the possibility of intensive care. Moreover, facilitating access to means favors the occurrence of SA^(27-28)^, as seen in this sample, with access to medicines, used by family members (p<0.001), and poisons within the home itself. The excessive use of antidepressants (p=0.001) and antipsychotics (p=0.018) was associated with their use in the treatment of a previous mental health problem, becoming a means for committing suicide. Furthermore, we observed that despite this important risk factor and cause, 27.6% of adolescents started using psychotropic drugs after being treated in the emergency room, a scenario that deserves special attention regarding the prescription guided and monitored by health professionals, in order to avoid recurrences.

It is noteworthy that in 27.6% of the consultations listed in this study there was the introduction of medication and 31.1% of psychotherapy. This data reflects the logic of care that still permeates mental health, with the early medicalization of care, using medication as a way to treat emotional aspects without reflecting the whole context of suffering in which the individual is inserted. In addition to this, the indication of psychotherapy becomes a classic example of referral of care, where, frequently, adolescents must seek in their health network places that provide this type of care autonomously, due to the difficulty of translating and accessing the policies of the mental health care network.

In a systematic review with meta-analysis that aimed to assess the magnitude and identify gender-specific risk and protective factors in SA and death by suicide in adolescents and young adults, it was observed that women are at a higher risk of SA and men at a higher risk of death by suicide. This finding is related to men’s greater access to violent means, such as firearms, pesticides and toxic gases^(29)^. This aspect corroborates with this study that 83% of adolescents were female, however stab wounds were means used only by male adolescents (p=0.02) as well as precipitation (p=0.02). Some studies^(27-28,30)^ associate this difference with greater exposure to risk factors by men, such as impulsivity, aggressiveness towards themselves and their family members and use of psychoactive substances.

As far as SA is concerned, an association can be made with a building block, in which when the structures are not firm, a new weight can lead to collapse. In this case, the structures are the risk and protection factors, and because SA is a multidetermined phenomenon, there may be an association of more than one risk factor and/or motivation by adolescents. Previous SA is the main risk factor for a future suicide^(27-28)^. In this study, previous SA was observed in 15% of cases. In a population-based, longitudinal study conducted in Sweden, it is observed that, of 13,852 adolescents, 276 reported AS once, 116, between 2 and 3 times, 37, between 4 and 9 times, 19, over 10 times and 22, more times than they can count. Identification as a sexual minority was associated with a ten times greater chance of SA in adolescence (p<0.05), mainly due to the experience of stigma, concealment of identity and homophobia^(9)^. In this study, homosexuality was associated with a higher risk of SA in male adolescents (p<0.001).

In a retrospective cohort conducted in Canada between 2014 and 2015, it was observed that individuals with mental health problems had more visits to the emergency department. However, the outpatient clinic, for those who were followed up, became a protective factor against SA^(31)^. In an ideal health system, all adolescents at risk or with previous SA would be monitored in the services that make up the Psychosocial Care Network; however, in clinical practice, it is observed that the referral system does not reach the idealized longitudinality. Furthermore, it is worth reflecting that monitoring is not a guarantee of qualified care and problem-solving.

In March 2020, the WHO decreed the COVID-19 pandemic, consequently the number of visits to the emergency and emergency departments for children were reduced. However, regarding the risk of suicide in a systematic review with meta-analysis^(13)^, which aimed to systematize the evidence on suicide, self-mutilation and suicidal ideation during the COVID-19 pandemic, it was observed that both the direct and indirect effects of the pandemic can increase SA rates, which the prevalence in this period was 11, 5% in the general population. This aspect corroborates a retrospective cross-sectional investigation, carried out in Texas, in an emergency service with adolescents, which an increase in SA is observed, when comparing the year 2020 to 2019 (p<0.05), the chances of suicidal ideation were higher between the months of March (OD: 1.6) and July 2020 (OD: 1.4) compared to 2019^(14)^.

This increase may be associated with the potentialization of risk factors, such as isolation, economic recession, stress, lack of access to educational and health resources^(13)^, and greater exposure to the social determinants exposed in this study, such as physical, psychological and/or sexual abuse, conflicts and grief for loved ones who may have died from COVID-19. This aspect goes hand in hand with the fact that there was a greater restriction on emergency services, main gateway for individuals with SA, and in specialist mental health secondary-level services, which can reduce the search for care and accentuate suicidal behavior.

In this study, data were not analyzed by trend, which limits us from confirming whether there was a reduction or increase in the demand for SA services during the pandemic. Another aspect is that the data were collected only until July 2020, not being able to be compared with previous data, however, there are seven consultations in seven months, which is a considerable number.

It is estimated that SA exceeds the number of suicides by at least ten times, but the data are still unreliable^(15)^, mainly due to underreporting, as seen in this study, which only 26.1% of cases were reported, despite the notification is mandatory since 2010^(8-9)^. In hospital services, as seen in this sample, there is the use of a variety of codes and classifications related to SA, such as poisoning and accidents, which do not necessarily illustrate the intention of suicide at any stage of care, leading to consequences on the actual statistics of this event in health services. Another point that can mask SA and suicide are accident reports, since in Brazil mortality rates portray the injury that caused death and not the intention^(15)^.

In order to change this context, the WHO released in 2021 the “Live-Life” guide, which brings possible strategies for suicide prevention, with a focus on ensuring intersectoral collaboration; population awareness; professional training; funding of services that work with prevention; surveillance and monitoring of individuals at risk; and assessment to identify individuals with suicidal behavior^(32)^.

As an assessment possibility, in a multicenter randomized clinical trial carried out in 8 emergency services in the United States, which aimed to implement suicidal behavior assessment scales and telephone longitudinal follow-up, it was observed that, out of 236,791 visits to the service, 10,625 adolescents with this behavior were identified, and the data increased significantly throughout the implementation of this study (p<0.001), mainly with screening in all consultations (p<0.001), and there was an increase in documentation from 2.9% to 5.7%^(11)^. In the emergency department of this study there is no such triage, but this investigation demonstrates a new possibility of action.

Another point is that several studies point out in their conclusions^(3,10)^ that there is a need for new strategies and public policies to assist suicidal behavior. However, the WHO includes the reduction of suicide mortality as a global goal and as an indicator of the United Nations Sustainable Development Goals, as well as in the Comprehensive Mental Health Action Plan by 2030, bringing the “Live-Life” guide as a strategy^(32)^. In addition to this, in Brazil there have been policies since 2006^(3)^.

The main issue is not the formulation, but the feasibility and possibility of implementation in clinical practice in a context of lack of investment in health resources; retreat of mental health care; care based on medicalization and referral; health professionals who undervalue emotional, social and spiritual aspects, and overvalue clinical care; and health services unprepared for holistic care for mental health problems.

Thus, it is worth reflecting: how to ensure that the service network is organized, interconnected, communicating and qualified to provide assistance to SA? How to make health services welcoming spaces for adolescents with suicidal behavior? How to enhance protective factors in an environment where wear is prevalent? In this context, so that adolescents with suicidal behavior can be assisted with quality in health services, it is necessary to change based on interconnection, with satisfactory communication between the networks that make up mental health policies and truly interprofessional and intersectoral care, providing satisfactory and longitudinal interpersonal relationships, which escape the logic of referral, counter-referral and centralized care. To this end, nurses working in the emergency service can start with the basics of this line of reasoning: embracement, as long as it is sincere, allows the beginning of adolescents’ reconnection with their protective factors^(22)^.

### Study limitations

This study was carried out only in one institution with a retrospective design, being limited to records made by health professionals, which probably do not reflect the care provided in its entirety. There was difficulty in capturing the medical records due to outsourcing their conservation and location; underreporting of SA as well as the variety of codes used for SA and suicide; the care provided by different teams, with pediatrics at AER and medical-surgical clinic at AER, which had repercussions on the low level of detail of identified risk factors and quality of records.

### Contributions to nursing, health, or public policies

This study demonstrates the need for prevention, assistance, notification, longitudinal care with integration between networks, to be a priority topic in the health, education and social assistance agendas, with permanent education and interdisciplinary action, in order to expand the boundaries of interpretation of the phenomenon, not only in emergency departments, not circumscribing it, but recognizing it in its plural dimensions.

## CONCLUSIONS

In this study, it is observed that the emergency department deals with cases of suicidal behavior, with adolescents exposed to a context of multiple risk factors that culminate in SA, with intoxication prevailing, as a possibility of relief from suffering. In care, there are multiple repercussions, interventions and hospitalizations with a focus on clinical and medicalization, and possibly failure in reception. Over the years, an average has been observed in the number of visits, but it is known that the phenomenon is much greater, but there are still underreporting and classifications of the service without explicit intentionality.
